# *Antrodia camphorata* Grown on Germinated Brown Rice Suppresses Melanoma Cell Proliferation by Inducing Apoptosis and Cell Differentiation and Tumor Growth

**DOI:** 10.1155/2013/321096

**Published:** 2013-02-25

**Authors:** Minjung Song, Dong Ki Park, Hye-Jin Park

**Affiliations:** Department of Bioscience and Biotechnology, Konkuk University, Gwangjin-gu, Achasan-ro 263, Seoul 143-701, Republic of Korea

## Abstract

*Antrodia camphorata* grown on germinated brown rice (CBR) was prepared to suppress melanoma development. CBR extracts were divided into hexane, EtOAc, BuOH, and water fractions. Among all the fractions, EtOAc fraction showed the best suppressive effect on B16F10 melanoma cell proliferation by CCK-8 assay. It also showed the increased cell death and the changed cellular morphology after CBR treatment. Annexin V-FITC/PI, flow cytometry, and western blotting were performed to elucidate anticancer activity of CBR. The results showed that CBR induced p53-mediated apoptotic cell death of B16F10. CBR EtOAc treatment increased melanin content and melanogenesis-related proteins of MITF and TRP-1 expressions, which supports its anticancer activity. Its potential as an anticancer agent was further investigated in tumor-xenografted mouse model. In melanoma-xenografted mouse model, melanoma tumor growth was significantly suppressed under CBR EtOAc fraction treatment. HPLC analysis of CBR extract showed peak of adenosine. In conclusion, CBR extracts notably inhibited B16F10 melanoma cell proliferation through the p53-mediated apoptosis induction and increased melanogenesis. These findings suggest that CBR EtOAc fraction can act as an effective anticancer agent to treat melanoma.

## 1. Introduction

Melanoma is the most serious type of skin cancer, and recently it has become a leading cause of death among the various skin diseases. For example, about 48,000 melanoma-related deaths occur every year on a worldwide basis [1, 2]. Moreover, the incidence of melanoma has steadily increased in the past few decades [3]. When diagnosed with malignant melanoma, most patients ultimately die of their disease within two years. As a malignant melanoma therapy, standard cancer therapies such as irradiation, chemotherapy, and surgical excision are applied. However, high resistance, limited efficacy, and side effects of current therapeutical methods result in a poor survival rate. Therefore, application of therapeutic agents from natural sources to patients has been attempted as an alternative treatment. Recent reports showed efficacy of several compounds from nutritional sources such as genistein and resveratrol against melanoma [4–6]. In these reports, although they have shown an inhibitory effect against melanoma tumor development and growth, some of them remain as chemoprevention rather than chemotherapy or only focus on *in vitro* study. Therefore further study is required for improving therapeutic efficiency and applying to the clinics. 

Herein, we focused on Changji mushroom (*Antrodia camphorata*) grown on germinated brown rice (CBR) as an anticancer agent against melanoma. As a traditional oriental medicine, *Antrodia camphorata* (AC) has been used to treat food and drug detoxification, diarrhea, abdominal pain, and hypertension. Recently, anticancer activities of *Antrodia camphorata* against human colon and breast cancer cells have been reported [7–9]. In addition, they induced an apoptosis of human ovarian cancer and hepatocellular carcinoma cells [10, 11]. However, the anticancer activity of *Antrodia camphorata* on melanoma has not been investigated yet.

In this study, Changji mushroom (*Antrodia camphorata*) on germinated brown rice (CBR) extracts was prepared. Its inhibitory effect against B16F10 melanoma cell proliferation was evaluated. The cause of cancer cell death was further investigated with annexin V-FITC/PI staining, flow cytometry, the measurement of melanin content, and western blot analysis. In addition, CBR extract was administered to melanoma xenografted mice to investigate its inhibitory effect on melanoma growth. 

## 2. Materials and Methods

### 2.1. Cell Culture

B16F10 mouse melanoma cell line was purchased from American Type Culture Collection (Rockville, MD, USA) and cultured in minimum essential media (MEM) (Invitrogen, Carlsbad, CA, USA) supplemented with 10% fetal bovine serum (FBS) (Invitrogen) and 200 U/mL penicillin-streptomycin (Sigma, St. Louis, MO, USA). In all experiments, cells were grown to 80%–90% confluence and subjected to no more than 15 passages.

### 2.2. Preparation of CBR Extract

*Antrodia camphorata* on germinated brown rice (CBR) was provided by Cell Activation Research Institute (CARI, Seoul, Korea). Authenticated voucher specimens of *Antrodia camphorata* (AC) (Kucari 1101) and CBR (Kucari 1102) are deposited in the Herbarium at College of Bioscience and Biotechnology, Konkuk University (Seoul, Republic of Korea). AC was inoculated on germinated brown rice and cultured at 20–25°C for 4 weeks. Powder was extracted under reflux with 80% MeOH. The powdered material (1 kg) was extracted under reflux with 80% EtOH. The total extract (178 g, yield [w/w], 17.8%) was dissolved in water. After removing the insoluble solid particles by filtration, the liquid phase was extracted sequentially by solvents with increasing polarity (hexane, EtOAc, BuOH, and water; 1 : 10 [w/v] for all solvents) to yield four fractions. The liquid-liquid phase extraction was performed in Erlenmeyer flasks by shaking, and the extracts were concentrated to dryness by a rotary evaporator. Thus, we obtained the following fractions: hexane fraction (16 g, yield (w/w) 1.6%), EtOAc fraction (4.5 g, yield (w/w) 0.45%), BuOH fraction (8.25 g, yield (w/w) 0.825%), and water fraction (10.86 g, yield (w/w) 1.086%).

### 2.3. Cell Proliferation Assay

Melanoma cell viability in the absence and presence of different concentrations of AC and CBR was measured with CCK-8 assay (Dojindo, Rockville, MD, USA). Briefly, cells were plated onto 96-well plates (5 × 10^3^ cells/well) and treated with CBR (0, 1, 10, and 25 *μ*g/mL) for 48 and 72 hours. CCK-8 solutions were added and incubated for 2 hours, followed by viable cell detection with a microplate reader at 450 nm absorbance (Model 550; Bio-Rad, Hercules, CA, USA).

### 2.4. Annexin V-FITC/PI Staining

The extent of B16F10 apoptosis by CBR EtOAc fraction was evaluated by annexin V-FITC/PI apoptosis detection kit (Trevigen Inc., Gaithersburg, MD, USA), according to the manufacturer's instructions. B16F10 melanoma cells were incubated in a 6-well plate (3 × 10^5^ cells/well) and treated with CBR EtOAc fraction at different concentrations (0, 1, 5, and 10 *μ*g/mL) for 48 hours. Cells were harvested and fixed in ice-cold 70% ethanol at 4°C overnight. FACS was used to determine the percentage of cells displaying annexin V-FITC^+^/PI^+^ (necrosis), annexin V-FITC^−^/PI^+^ (late apoptosis), annexin V-FITC^−^/PI^−^ (normal), or annexin V-FITC^+^/PI^−^ staining (early apoptosis). At least 10,000 cells were used for each analysis, and experiments were performed in triplicate.

### 2.5. Measurement of Melanin Content

B16F10 melanoma cells were placed in a 24-well plate (8 × 10^4^ cells/well) in phenol-red-free media and incubated with CBR EtOAc fraction at 0, 1, 5, and 10 *μ*g/mL concentrations for 48 hours. The adherent B16F10 cells were washed with PBS and detached using 0.05% Trypsin-EDTA. Cells were collected in a test tube and washed twice with PBS. The melanin was then extracted using a mixture of 1 N NaOH: 10% DMSO at 80°C for 1 h. After centrifugation at 3000 rpm for 5 min, the melanin content was determined at 475 nm using an ELISA microplate reader.

### 2.6. Western Blot Analysis

B16F10 melanoma cells were lysed in RIPA buffer (1% Triton X-100, 100 mM Tris-HCl, pH 7.5, 10 mM NaCl, 10% glycerol, 1 mM sodium orthovanadate, 50 mM sodium fluoride, 1 mM *p*-nitrophenyl phosphate, 1 mM phenylmethylsulfonyl fluoride, and protease inhibitors, all from Sigma). Protein concentration was measured with the Bradford protein assay reagent (Bio-Rad), and equal amounts of proteins were applied to 12% SDS-PAGE gel. Proteins were transferred to nitrocellulose membrane (Millipore; Billerica, MA, USA) and blocked in 5% nonfat milk for 1 hour. Samples were probed with the following primary antibodies: p53, microphthalmia-associated transcription factor (MITF), TRP-1, and *β*-actin (all from Santa Cruz, Santa Cruz, CA, USA). Secondary antibodies were horseradish peroxidase conjugated goat anti-rabbit or anti-mouse antibodies (Pierce, Rockford, IL, USA). Enhanced chemiluminescence reaction was performed using a SuperSignal West Femto enhancer kit (Pierce), and the positive bands were detected on X-ray film.

### 2.7. Tumor Growth Analysis *In Vivo*

The inhibitory effect of CBR EtOAc fraction on melanoma tumor growth was investigated in an animal model. C57BL/6 mice (6 weeks, female) were purchased from Dae-Han Experimental Animal Center (Eumsung, Korea). All animals were handled following the guidelines of the Institutional Animal Care and Use Committee (IACUC) at Konkuk University (Seoul, Republic of Korea). Tumors were induced on the skin as a melanoma mouse model as previously described [12]. Experiments were performed in four groups: normal control-saline injection; tumor control-B16F10 cell (2 × 10^5^ cells/mouse) injection intraperitoneally; CBR group-B16F10 and Changji *(Antrodia camphorata)* grown on germinated brown rice (CBR) EtOAc fraction injection by IP; Dox group-doxorubicin (Sigma) 1 mg/kg/day (*n* = 7 per group). In CBR and Dox groups, CBR EtOAc fraction (100 mg/kg/day) and doxorubicin (1 mg/kg/day) administration started three days before B16F10 melanoma cell transplantation until sacrifice. Body weight was measured every three days. Tumor was analyzed on day 15 following transplantation of B16F10 melanoma cells. Mice were sacrificed 15 days following cell inoculation, and morphology of tumor growth mass was imaged with digital camera (Power Shot A470; Canon, Tokyo, Japan), and tumor viscera were collected for histology and weight analysis.

### 2.8. HPLC Analysis

In order to analyze the compounds in the extracts, high performance liquid chromatography (HPLC) experiments were carried out on Agilent 1260 Infinity HPLC system (Santa Clara, CA) with the reversed phase column (Luna C18, 250 × 4.6 mm, 5 *μ*m diameter, Phenomenex, Torrance, CA). The flow rate and injection volume were 1.2 mL/min and 5–20 *μ*L, respectively. The chromatograms were detected at 260 nm and collected at 30°C. Adenosine was purchased from Sigma-Aldrich, St. Louis, MO, 99%, and used as an authentic standard. One mg of adenosine was dissolved in 1 mL of 50% ethanol and filtrated using 0.45 *μ*m membrane filters. While the mobile phase for the ethanol extract of CBR was 8% aqueous methanol, that for other fractions separated based on the hydrophobicity was 6% aqueous methanol containing 0.1% KH2PO4.

### 2.9. Statistical Analysis

Data are expressed as means ± standard error of the mean (SEM). Statistical analyses were performed using Student's *t*-test in Excel program (Microsoft, Redmond, WA, USA). Significant differences were considered from *P* < 0.05.

## 3. Results

### 3.1. *Antrodia camphorata* Grown on Germinated Brown Rice (CBR) Fraction Inhibits B16F10 Melanoma Cell Proliferation

**Figure 1:**
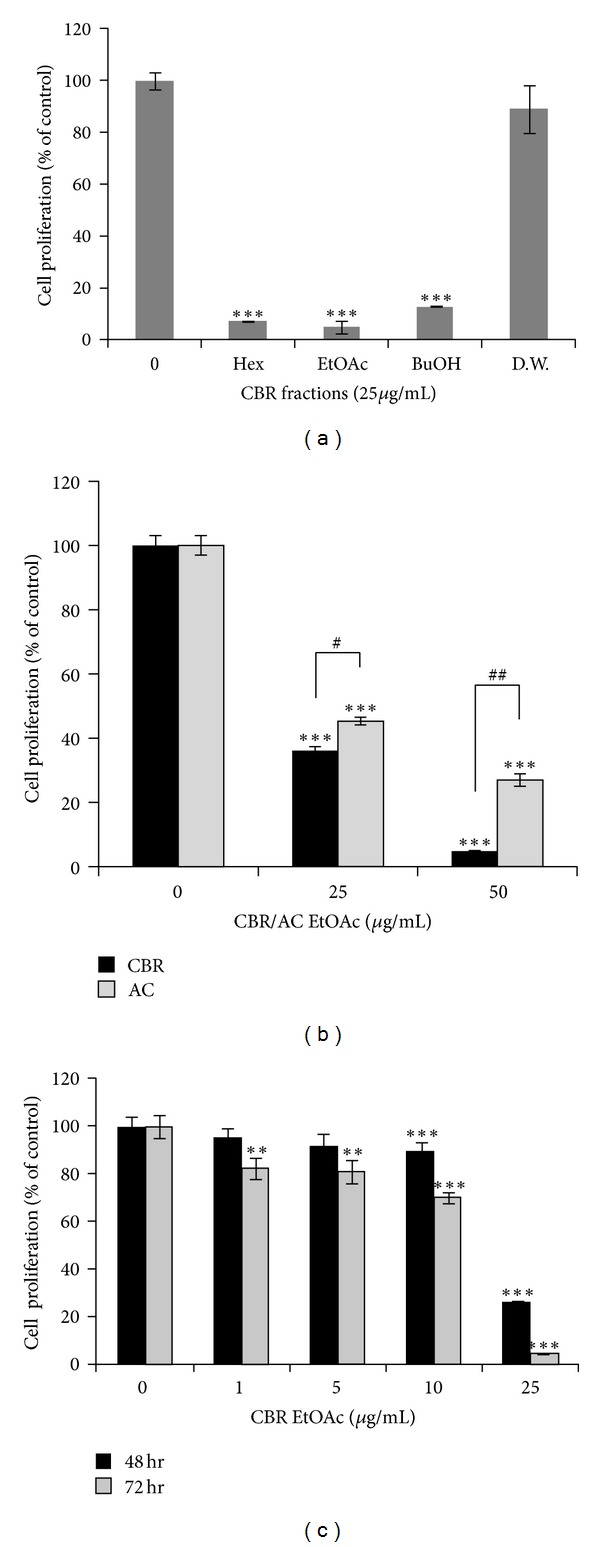
(a) B16F10 melanoma cell proliferation at 72 hours in the presence of each CBR fraction (hexane, EtOAc, BuOH, and water) compared to control. (b) Cell proliferation on CBR and AC EtOAc fraction (0, 25, and 50 *μ*g/mL) for 48 hours (^#^ *P* < 0.01, ^##^ *P* < 0.001). (c) B16F10 melanoma cells were treated with CBR EtOAc fraction at various concentrations (0, 1, 5, 10, and 25 *μ*g/mL) for 48 hours. Cell viability was compared to control, and statistically different level was denoted by *P < 0.05, **P < 0.01, ***P < 0.001.

 To determine the effect of *Antrodia camphorata* grown on germinated brown rice (CBR) fractions on B16F10 melanoma cell growth, we performed a cell proliferation assay as a screening tool for its anticancer activity. CBR hexane and BuOH fractions at 25 *μ*g/mL inhibited B16F10 cell proliferation, with the CBR EtOAc being the most potent in antiproliferative activity (Figure 1(a)). Among the three fractions, the EtOAc fraction has the most efficient suppressive effect. Distilled water fractions had no effect on melanoma cell proliferation. Antiproliferative activity of CBR and AC (*Antrodia camphorata*) EtOAc fraction against B16F10 cells was compared. The IC_50_ of CBR EtOAc fraction (IC_50_, 46.84 ± 1.53 *μ*g/mL) was significantly lower than that of AC EtOAc fraction (IC_50_, 60.20 ± 3.27 *μ*g/mL) (Figure 1(b)). This indicates that the inhibitory efficacy of CBR EtOAc fraction on melanoma cell proliferation is more effective than ordinary AC EtOAc fraction. Based on this data, we chose EtOAc fraction for further investigations of melanoma cell death.  Figure 1(c) showed B16F10 melanoma cell proliferation in the presence of different EtOAc fraction concentrations (0, 1, 5, 10, and 25 *μ*g/mL). B16F10 melanoma cell proliferation displayed dose-dependent decrease after CBR treatment. The IC_50_ value of CBR EtOAc fraction against B16F10 cell proliferation was calculated as 19 *μ*M and 16 *μ*M at 48 h and 72 h, respectively. These observations led to the selection of doses for further mechanistic and molecular studies that ranged from 1 to 20 *μ*M. CBR EtOAc fraction did not affect normal cell (Raw264.7 mouse macrophage) proliferation at 25 *μ*g/mL concentration.

### 3.2. CBR Induced Melanoma Cell Apoptosis

**Figure 2:**
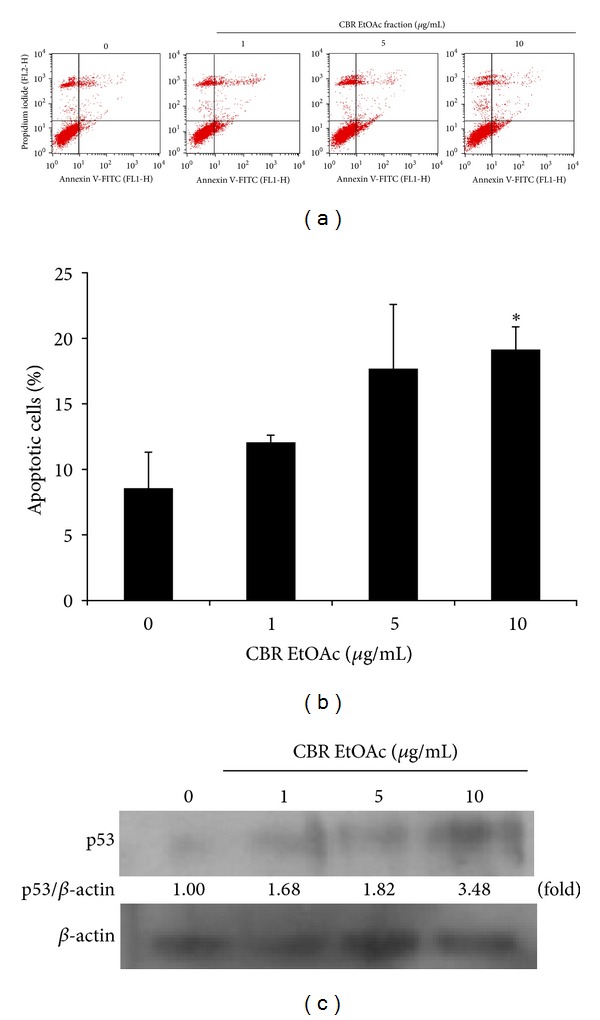
(a) Annexin V-FITC/PI staining analysis for apoptosis. Cells were treated with 1, 5, and 10 *μ*g/mL CBR EtOAc fraction for 48 hours. After treatment, the cells were stained with Annexin V-FITC/PI and subjected to flow cytometry analysis. (b) The quantitive result of apoptotic cells. Significant difference compared to the control is denoted by *P < 0.05. (c) p53 expression by western blotting on 0, 1, 5, and 10 *μ*g/mL CBR EtOAc treatment.

B16F10 cell death was further analyzed using annexin V-FITC/PI staining and flow cytometry (Figure 2). CBR EtOAc induced early (annexin V FITC^+^/PI^−^) and late (annexin V FITC^−^/PI^+^) apoptosis as shown in Figures 2(a) and 2(b). Data showed the dose-dependent increase in the numbers of apoptotic cells after 48 hr treatment of CBR EtOAc fraction. In the control group, 8.58% (early apoptosis + late apoptosis) were positive for annexin V-FITC staining, while CBR EtOAc fraction treatment resulted in 12.05%, 17.68%, and 19.13% at 1, 5, and 10 *μ*g/mL, respectively. The increased level of p53 protein expression was observed after 48 hour incubation with CBR EtOAc fraction. The p53 protein is a marker of apoptosis, known as an apoptosis initiation master switch [13, 14]. This result indicates that the inhibition of melanoma cell proliferation by CBR occurs via p53-mediated apoptosis. 

### 3.3. CBR EtOAc Fraction Induced Melanin Synthesis and the Expression of Melanogenesis Marker Molecules in B16F10 Cells

**Figure 3:**
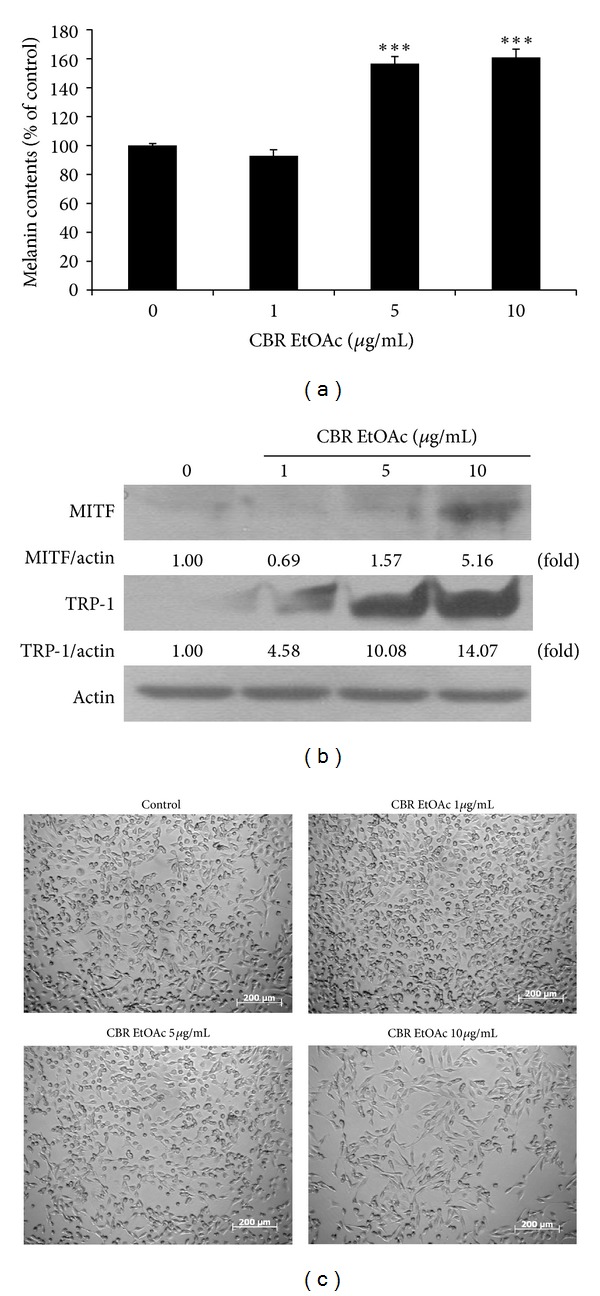
(a) Melanin production in B16F10 melanoma cells upon CBR EtOAc treatment (0, 1, 5, and 10 *μ*g/mL) for 48 hours. The melanin content was measured with a microplate reader at 475 nm. All experiments are performed in triplicate (***P < 0.001). (b) Representative western blot images of MITF, TRP-1, and *β*-actin under EtOAc fraction treatment (0, 1, 5, and 10 *μ*g/mL). (c) Phase contrast microscopy: representative images of B16F10 melanoma cells treated with 0, 1, 5, and 10 *μ*g/mL CBR EtOAc and controls for 48 hours (scale bar = 200 *μ*m).

Melanogenesis occurs in differentiated melanoma cell. There is a report that the differentiated melanoma is associated with slower cell proliferation [15]. We studied how the CBR EtOAc fraction affected melanogenesis of B16F10 cells. As seen in Figure 3(a), CBR EtOAc fraction induced the melanin synthesis in a dose-dependent manner (0, 1, 5, and 10 *μ*g/mL). To demonstrate the detailed mechanism in melanin synthesis, the level of TRP-1 and MITF protein was investigated. MITF and TRP-1 have a crucial role in the survival and differentiation of melanoblasts and melanocytes [16, 17]. After CBR EtOAc treatment, the levels of TRP-1 and MITF proteins were increased (Figure 3(b)), which indicated processed melanogenesis. Next, we checked whether CBR EtOAc fraction affected cellular morphology (Figure 3(c)). In control group, above 90% confluence of B16F10 melanoma cells was observed, and they still keep spindle-shape morphology after 48 hours. In contrast, CBR EtOAc fraction-treated group exhibited relatively less number of cells, and more dendritic-like shaped cells were observed, which is a morphological indicator for melanoma cell differentiation [18, 19].

### 3.4. CBR Inhibits Xenografted Melanoma Tumor Growth

**Figure 4:**
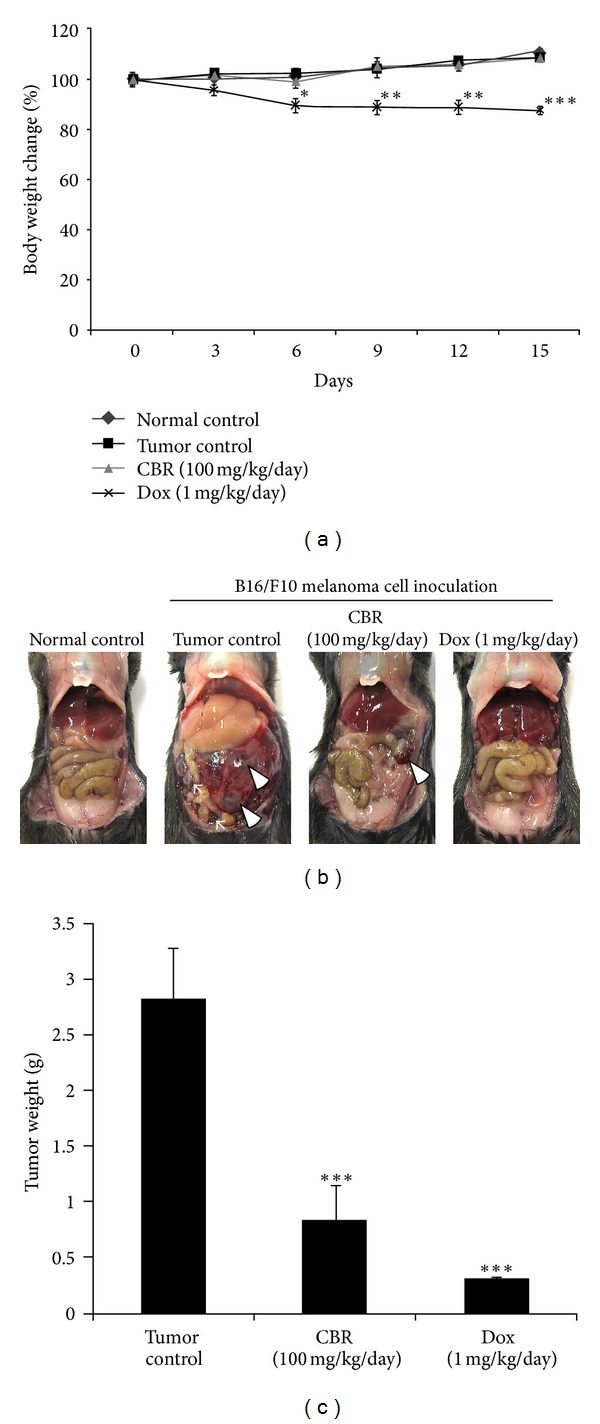
Effect of CBR EtOAc fraction on tumor growth in melanoma cell xenografted mice. (a) Average body weight of control and CBR, Dox-treated mice plotted over days after tumor cell inoculation: points: mean of seven animals. (b) Tumor nodule (arrow) and viscera mass (arrow head) of B16F10-inoculated mice treated with CBR (100 mg/kg/day) and Dox (1 mg/kg/day). (c) Tumor weight changes in B16F10-inoculated mice treated with CBR (100 mg/kg/day) and Dox (1 mg/kg/day) and controls (*P < 0.05, **P < 0.01, ***P < 0.001).

To investigate whether CBR EtOAc fraction has an inhibitory effect on the tumor growth in mice, CBR was intraperitoneally administrated to mice with melanoma. We examined the suppressing effects of CBR EtOAc fraction on peritoneally disseminated melanoma tumor growth. At 15 days following melanoma cell grafting, peritoneal tumor nodules were counted and weighed. Body weights of mice were recorded every 3 days during the experiments and are displayed in Figure 4(a). CBR feeding appeared tolerable as depicted by similar body weight compared to control groups. However, mice receiving doxorubicin (1 mg/kg/day) showed significant decrease (12.31 ± 1.68%) in body weights, compared to CBR EtOAc fraction-treated or control group (*P* < 0.05). At day 15th, melanoma was widely disseminated on whole intestine. In contrast, the peritoneally disseminated tumor mass growth was significantly inhibited in mice given CBR EtOAc fraction (100 mg/kg/day) (Figure 4(b)). In the tumor control group, mean of tumor weight amounted to 2.84 ± 0.45 g, whereas the mean of tumor burden in CBR EtOAc fraction-treated group was only 0.84 ± 0.30 g, representing a significant suppression in tumor growth by 29% (Figure 4(c)).

### 3.5. Chromatogram of Adenosine on CBR and AC

**Figure 5:**
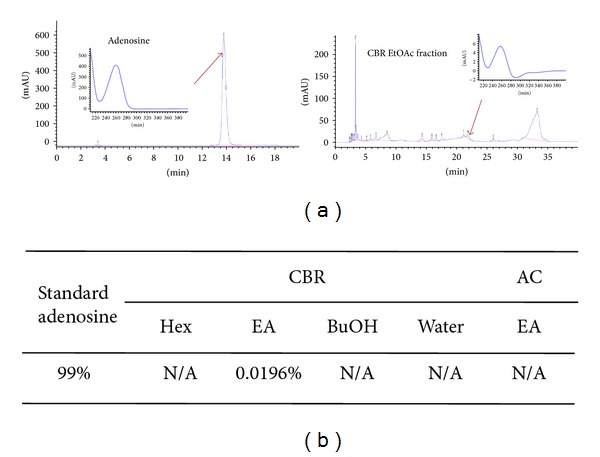
Representative high-performance liquid chromatography characterization of the components with detection at 230 nm showing adenosine (arrow) in adenosine standard and CBR extract.

In the HPLC experimental condition for the fractions separated based on the hydrophobicity, the authentic adenosine was observed at the retention time of 22 min in its chromatogram as 99%. Among the samples, adenosine was observed from CBR EtOAc fraction, and its concentration was 0.0196% (Figure 5), and it was not detected on AC extract and other CBR fractions (Hex, BuOH, and water). 

## 4. Discussion

Melanoma is the most serious type of skin diseases with many obstacles in traditional cancer therapy (e.g., resistance to current method and fast metastatic property). Therefore, establishing more effective and safe treatment regimen is in need. In this study, we investigated Changji mushroom (*Antrodia camphorata*) grown on germinated brown rice (CBR), as a novel and efficient antimelanoma agent. *Antrodia camphorata* (AC) is a well-known medicinal mushroom that has been used in oriental medicine for treating various diseases. Previous studies have demonstrated that AC has a wide range of pharmacological activities, including anticancer properties [7–11]. Specifically, AC induces apoptotic cell death in human leukemia [20], breast [9], ovarian [11], colon, [21] and liver cancer cells [10]. However, apoptotic effect of AC on melanoma cells has never been studied. In the present study, the inhibitory effect of AC on germinated brown rice (CBR) and on melanoma cell growth was investigated including the induction of apoptotic cell death and melanogenesis as well as the suppressive effect on melanoma growth with the xenografted mice. 

Cancer cells generally show uncontrolled/high proliferation, migration, and matrix-invasion potentials [22]. They lost the regulation of cell-cycle checkpoints or were resistant to the programmed cell death (apoptosis). Therefore, inhibition of tumor growth is the most attractive approach in developing anticancer agents. In Figure 1, B16F10 cell proliferation was significantly inhibited in the order of CBR-EtOAc fraction > CBR-BuOH fraction > CBR-hexane fraction. Therefore, CBR EtOAc fraction was chosen for further studies. 

Figure 2 results elucidated that the CBR EtOAc treatment increased p53-mediated apoptotic cell death. Apoptosis is a programmed cell death via the intricate pathways and is known to be deeply associated with the early stage of skin carcinogenesis [23]. Annexin V-FITC/PI staining was performed to confirm the apoptotic cell population increase with CBR EtOAc fraction treatment (Figures 2(a) and 2(b)). p53 protein is a transcription factor that being profoundly involved in apoptosis and used as a tumor suppressor was investigated [13, 24]. Increased level of p53 proteins under CBR EtOAc treatment demonstrated that p53 had a major role in apoptotic B16F10 melanoma cell death (Figure 2(c)). Similarly, Tokgun et al. reported that medicinal plants of *Convolvulus galaticus, Crocus antalyensis*, and *Lilium candidum* extracts showed cytotoxic effect on human breast cancer cells by p53-mediated apoptosis [25]. It seems that CBR EtOAc fraction exerts its antitumor activity through inducing the cellular apoptotic response. 

We also tested whether the CBR EtOAc fraction induced melanogenesis based on the recent report that the differentiated melanoma was associated with slower cell proliferation [15]. As seen in Figure 3, CBR EtOAc treatment significantly increased melanin production and MITF and TRP-1expression, which are melanogenesis-related proteins. Melanin synthesis occurs in the melanosome. MITF was a master regulator of its core component, and its upregulated protein expression resulted in cellular differentiation [16, 26]. TRP-1 is also an important melanosome core component [17]. Based upon these results, the suppressive effect of CBR EtOAc fraction against B16F10 melanoma cell proliferation can be explained as an increased apoptosis and an induced melanogenesis. 

In addition, the anticancer effect of CBR EtOAc fraction was confirmed in melanoma-xenografted animal model. Other previous studies that showed AC against colon or breast cancer demonstrated its anticancer activity only in the cellular level [7–9]. However, *in vivo* efficacy testing is a critical step for clinical application. We used 100 mg/kg of CBR EtOAc fraction, and significant melanoma growth suppression was observed compared to control (Figure 4). This CBR concentration is relatively low concentration compared to other studies. For example, when the anticancer activity of resveratrol was tested *in vivo*, dose of 40–1,500 mg/kg (RES/mice body weight) was applied [5, 27]. Using dose scaling as advised in FDA guidance, the human equivalent dose of CBR EtOAc fraction in our *in vivo* study can be applied to approximately 427.26 mg of CBR and to a 60 kg adult and represents a reasonable starting point for future clinical investigations. 

As seen in Figure 1, CBR EtOAc fraction showed more potent antimelanoma cell proliferative activity compared to AC EtOAc fraction and other CBR fractions. The active components of each CBR EtOAc fraction were analyzed by HPLC analysis, and only CBR EtOAc fraction contained adenosine. Adenosine showed an antitumor effect and was used as a potent regulator of normal and tumor cell growth [28, 29]. Recently, Madi et al. reported that adenosine increased melanin level by activating adenosine receptors [30]. 

## 5. Conclusion

Current study demonstrated that CBR EtOAc fraction inhibited melanoma cell proliferation through the induction of subsequent apoptosis and melanogenesis. Importantly, *in vitro* study result was reflected in our* in vivo* studies that showed significant melanoma growth inhibition in mice implanted with melanoma xenografts upon treatment with CBR EtOAc fraction. Adenosine was investigated as an active component from CBR EtOAc fraction. Further work will be focused on isolating active components in CBR EtOAc extract that contribute to its anticancer activity. In future applications, this study gives the strong evidence that CBR extract may be a strong candidate in novel cancer prevention and therapeutic strategies.
